# Human gene correlation analysis (HGCA): A tool for the identification of transcriptionally co-expressed genes

**DOI:** 10.1186/1756-0500-5-265

**Published:** 2012-06-06

**Authors:** Ioannis Michalopoulos, Georgios A Pavlopoulos, Apostolos Malatras, Alexandros Karelas, Myrto-Areti Kostadima, Reinhard Schneider, Sophia Kossida

**Affiliations:** 1 Cryobiology of Stem Cells, Centre of Immunology and Transplantation, Biomedical Research Foundation, Academy of Athens, Soranou Efessiou 4, Athens, 11527, Greece; 2 ESAT-SCD/IBBT-K.U. Leuven Future Health Department, Katholieke Universiteit Leuven, Kasteelpark Arenberg 10, Heverlee-Leuven, 3001, Belgium; 3 Department of Cell Biology and Biophysics, Faculty of Biology, University of Athens, Panepistimiopolis, Athens, 15701, Greece; 4 Wellcome Trust Genome Campus, European Bioinformatics Institute, Cambridge, CB10 1SD, United Kingdom; 5 Bioinformatics & Medical Informatics Team, Biomedical Research Foundation, Academy of Athens, Soranou Efessiou 4, Athens, 11527, Greece; 6 Structural and Computational Biology Unit, European Molecular Biology Laboratory, Meyerhofstrasse 1, Heidelberg, 69117, Germany; 7 Luxembourg Centre for Systems Biomedicine (LCSB), University of Luxembourg, Campus Belval, avenue des Hauts-Fourneaux 7, Esch sur Alzette, L-4362, Luxembourg

**Keywords:** Microarray analysis, Gene annotation, Gene coexpression, Functional annotation

## Abstract

**Background:**

Bioinformatics and high-throughput technologies such as microarray studies allow the measure of the expression levels of large numbers of genes simultaneously, thus helping us to understand the molecular mechanisms of various biological processes in a cell.

**Findings:**

We calculate the Pearson Correlation Coefficient (*r-*value) between probe set signal values from Affymetrix Human Genome Microarray samples and cluster the human genes according to the *r-*value correlation matrix using the Neighbour Joining (NJ) clustering method. A hyper-geometric distribution is applied on the text annotations of the probe sets to quantify the term overrepresentations. The aim of the tool is the identification of closely correlated genes for a given gene of interest and/or the prediction of its biological function, which is based on the annotations of the respective gene cluster.

**Conclusion:**

*Human Gene Correlation Analysis* (HGCA) is a tool to classify human genes according to their coexpression levels and to identify overrepresented annotation terms in correlated gene groups. It is available at: http://biobank-informatics.bioacademy.gr/coexpression/.

## Findings

Genomics refers to the comprehensive study of genes and their function. Recent advances in bioinformatics and high-throughput technologies such as microarray analysis lead to a revolution in our understanding of the molecular mechanisms underlying normal and dysfunctional biological processes. Microarray analysis has become an important tool for studying the molecular basis of complex disease traits and fundamental biological processes, by measuring the expression of thousands of genes in a single sample. The main approach for the assignment of biological functions to genes, using microarrays, is the differential approach, where two or more sets of microarray results (*e.g.* from normal and diseased tissue) are compared and genes implicated can be pinpointed by their differential expression levels. In co-expression experiments, many microarrays, from various tissues or developmental stages of the same organism and under different experimental conditions, are combined using clustering techniques. With this approach one can group genes, which show correlated expression patterns, implying that those genes, are involved in connected biological processes. The co-expression of genes revealed in large databases of related and unrelated microarray experiments can contain information far beyond the original purposes for which the constituent experiments were performed, and can be a valuable predictive tool for gene function and pathway membership.

A wide range of gene coexpression analysis tools already exists; most of them are organism specific. The Arabidopsis Co-expression Tool, ACT [1,2], ranks the genes of *Arabidopsis thaliana* plant across a large microarray dataset consisting of ~21,000 genes from around 1400 arrays. A similar approach is adopted by Expression Angler [3]. As opposed to ACT which stores pre-calculated correlation data, Expression Angler produces them on the fly depending on the selected subset of data. This tool also identifies potential *cis*-regulatory elements in the promoters of co-regulated genes. ATTED-II [4,5] is a *trans*-factor and *cis*-element prediction database for *Arabidopsis thaliana* plant that provides co-regulated gene relationships based on co-expressed genes deduced from microarray data and the predicted *cis* elements. Genevestigator [6] analyses expression profiles from more than ~22,000 *Arabidopsis thaliana* genes including many uncharacterised genes. CSB.DB [7] currently focuses on *Escherichia coliSaccharomyces cerevisiae* and *Arabidopsis thaliana* model organisms. A comparative analysis [8] of those coexpression tools demonstrates the opportunities for hypotheses generation in plant biology.

In animal biology, SymAtlas [9] uses custom arrays that interrogate the expression of the vast majority of protein-encoding human and mouse genes to profile a panel of 79 human and 61 mouse tissues, whereas COXPRESdb [10,11] is mostly focused on comparing human and mouse genes between each other. Human gene coexpression landscape [12] constructs a coexpression network to allow further analyses on the network and on some specific gene associations. Another coexpression analysis [13] across 60 heterogeneous human experimental datasets was performed to establish a high confidence network of coexpressed genes found in multiple observations. Each data set is pre-processed and analysed to identify pairs of genes that are strongly coexpressed. A major upgrade of that tool is Gemma [14], a resource for re-use and meta-analysis of gene expression profiling data. Gemma contains data from ~3300 public microarray data sets, including over 1400 human ones. GEO DataSet cluster analysis visualisation tool [15] displays cluster heat maps. It contains datasets from all Affymetrix platforms, including 199 datasets from the GPL570 platform. WGCNA [16] is a comprehensive collection of R functions for performing various aspects of weighted correlation network analysis. While the current software was not applied to analyse our data, human related studies based on it were cited in our manuscript. Such studies involve analysis of data from brain cancer [17], diabetes [18-20], primate brain tissues [21] and chronic fatigue patients [22]. Finally, data integration and visualisation techniques have rapidly evolved in performing gene expression analysis by trying to bridge the gap between analysis and visualisation. Most notable examples are BioGPS [23] which is a centralised gene portal for aggregating distributed gene annotation resources, and BioLayout Express^3D^[24] which is able to provide visually 3D clusters of closely correlated bioentities. Most of the aforementioned approaches together with others [25-30] are mostly focused on expression data whereas more recent studies move towards data integration and knowledge management by combining microarray data with other data types, such as yeast-two-hybrid experiments, protein-protein interactions, literature co-occurrences or knowledge networks [31-34].

Here, we illustrate the features of the *Human Gene Correlation Analysis* (HGCA) tool, which is focused on a large-scale human gene correlation analysis. By pooling human microarray data from various tissues or developmental stages, we measure the coexpression levels between ~54000 probe sets across the human genome and subsequently cluster them, using a hierarchical clustering approach. HGCA groups genes with correlated expression patterns, implying that those genes are involved in connected biological processes. In addition, HGCA performs an analysis to find overrepresented terms that can give evidence about the underlying biological functions and processes. As such, HGCA tool can be used as a prediction tool for gene characterisation.

## Methods

### Data preparation and integration

For the purposes of our analysis, we extracted expression data from many Affymetrix Human Genome U133 Plus 2.0 Array Chip samples, as follows:

Using a PHP script, Simple Omnibus Format in Text (SOFT) files from samples of GPL570 or alternative platforms found in GEO repository [15] were downloaded and parsed. Title and characteristics were searched for keywords such as “healthy”, “normal”, “tissue” or “control” and sample organism for “*Homo sapiens*”. Additionally, all normal samples from manually curated human clinical microarray database M^2^DB [35] were selected, as follows: From M^2^DB website, we only selected individual samples of Human U133 plus 2.0 platform, applying no further quality control filtering. From them, only “Normal” GEO samples were chosen from “Disease State” clinical characteristics.

The annotations of all selected samples were manually read and only samples of healthy individuals or normal tissues adjacent to pathological ones were kept. Samples from cultured cell lines, pathological tissues or pharmacologically treated individuals were excluded. Each sample was manually classified according to the name of its tissue or organ.

After downloading the raw intensity files (CEL) of the chosen samples from GEO, a quality control was conducted using a PHP script which checked the raw files for errors in the probe intensity values. While the majority of files were in CEL version 3 ASCII format, a substantial number of them were in CEL version 4 binary format and had to be converted to the previous version format with apt-cel-converter, a program from Affymetrix Power Tools (apt-1.14.4) Software Package [36]. A PHP script parsed all ASCII CEL files and checked every intensity value of the 1164x1164 probes of each chip for being within the acceptable value range (0–65535). The script also concatenated all probe intensity values into a single string for each chip. This string was used as input for MD5 (RFC 1321), SHA-1 (RFC 3174) and CRC32 [37] hash algorithms and their outputs were concatenated as a single string, which then served as a characteristic signature of the probe intensities of each sample, in order to filter out duplicate GSMs. A list of unique GSMs was produced and a PHP script selected samples as evenly as possible, amongst all tissues/organs and GSE sample series.

To generate a single value that reflects the amount of each transcript in solution which corresponds to a probe set, apt-mas5, the Affymetrix Power Tools implementation of MAS5.0 algorithm [38], was used with the default Affymetrix Chip Description File (CDF) (HG-U133_Plus_2.cdf). Apt-mas5 output files (CHP) were converted to ASCII with the use of apt-chp-to-txt converter from Affymetrix Power Tools suite. Then, the data were normalised to allow different samples to be comparable, as follows: AFFX-prefixed control probe sets were excluded from the analysis and the rest of the 54613 probe sets were trivially normalised using the Affymetrix standard procedure where all signal values were multiplied by a scaling factor which was calculated by removing the top and bottom 2% of signal values, then calculating a value that adjusts the mean of the remaining 96% to 500. Finally, all signal values were rounded to the nearest 0.5.

Each probe set in our database was enriched with annotations collected from various data sources: Genomic data, such as HUGO Gene Nomenclature Committee gene symbols and descriptions, were collected from ENSEMBL [39], GO terms from the Gene Ontology Database [40], Enzyme Commission (EC) numbers and pathway information from KEGG [41], protein signature data from InterPro [42], genetic phenotypes from OMIM [43,44] and predicted *cis* element information by combining TransFac [45] and ENSEMBL [39] data.

### Promoter analysis

Regulatory sequences from 500 bps upstream of the Transcription Start Sites (TSSs) of all genes were collected from ENSEMBL [39] and were compared against the Transcription Factor Position-Weight Matrices (PWMs) from TransFac [45] using the MATCH algorithm [46] which is a weight matrix-based tool for searching putative transcription factor binding sites in DNA sequences. In our case, core and matrix similarity cut-offs were set to 0.95 and 0.90 respectively to increase stringency.

### Statistical analysis

The Pearson correlation coefficient (*r*-value) between two probe sets is defined as the covariance of the two probe sets divided by the product of their standard deviations and it is calculated as follows:

(1) $$r_{x,y}=\frac{\sum_{i=1}^{n}(x_{i}-\bar{x})(y_{i}-\bar{y})}{\sqrt{\sum_{i=1}^{n}{\left(x_{i}-\bar{x}\right)}^{2}\sum_{i=1}^{n}{\left(y_{i}-\bar{y}\right)}^{2}}}$$

where *r*_*x,y*_ is the Pearson correlation coefficient, *n* is the number of microarray experiments and *x*_*i*_ and *y*_*i*_ are the signal intensities of probe sets *x* and *y* in the *i*th experiment. *r-*values range between −1 and +1; positive *r-*values correspond to correlated probe sets, negative values to anti-correlated probe sets and values close to zero to non-correlated. A computationally efficient way to interpret Pearson correlation is to express it as the mean cross-product of the standardised variables [47]:

(2) $$r_{x,y}=\frac{\sum_{i=1}^{n}z_{x_{i}}z_{y_{i}}}{n}$$

where $z_{x_{i}}$ and $z_{y_{i}}$ are the standardised variables of the signal intensities of probe sets *x* and *y* in the *i*th experiment.

Assuming that the association between expression profiles is approximately linear, *t* which is distributed in the null hypothesis (of no correlation) like Student’s *t-*distribution with *ν = n-*2 degrees of freedom, can be calculated as follows [48]:

(3) $$t_{x,y}=r_{x,y}\sqrt{\frac{\nu }{1-r_{x,y}{}^{2}}}$$

Its two sided significance level *p*_*x,y*_, is given by Student’s Distribution Probability Function [49]:

(4) $$p_{x,y}=A(t_{x,y}|v)$$

To account for multiple sampling, *p-*values were Bonferroni corrected [50], as follows:

(5) $$\begin{array}{c} np_{x,y}\leq 1:e_{x,y}=np_{x,y}\\ np_{x,y}>1:e_{x,y}=1 \end{array}$$

where *e-*values are Bonferroni corrected *p-*values. The pairwise *r-* and *e-*values were stored in a MySQL database.

### Clustering analysis

We created a symmetric correlation matrix *R(x,y)* between all probe sets stored in the database. The all-against-all correlation matrix has size *m*x*m* where *m* = 54613 is the number of probe sets. We expressed the network as a distance matrix *D(x,y)* where each value is calculated as *D(x,y) = 1-R(x,y)*. The distance matrix data were stored as a Phylip format file [51] and we applied Neighbour Joining algorithm (NJ) [52] to cluster the data. The algorithm takes as input the Phylip file and constructs a rooted hierarchical tree in Newick format. NJ algorithm is computationally efficient because of its polynomial-time complexity [53] and thus can be applied on very large data sets. We chose the Quick Join [54] implementation which uses heuristics for speeding up the NJ algorithm while still constructing the same tree as the original algorithm.

### Implementation

We setup a PHP-based web site for HGCA, which allows interactive searches for gene names, probe sets or annotation terms. The interface allows querying for two complementary questions. Users interested in a particular probe set can retrieve: a) an *r-*value-ranked list of the most closely correlated probe sets, b) a tree-based list of the most closely clustered probe sets.

To simplify the navigation, the web interface was designed as simple as possible in a way that makes the navigation friendly and the extraction of knowledge easy, producing a tool that can be used by any experimentalist. The colour scheme allows the easier understanding of information and the simplification of human computer interaction. Thus, lines coloured pink highlight the probe sets that refer to the query gene whereas the green lines highlight the collected co-expressed probe sets to the query gene. The grey lines indicate that the co-expressed probe set appear in the list but the gene for the specific probe set was previously highlighted in the list by another co-expressed probe set.

The tree-based clustering will organise the most closely correlated probe sets to the driver gene in a tree hierarchy. The trees can be either visualised within the HTML web page or downloaded as Newick files to be visualised by external applications [55]. The interactive interface allows adjusting the height of the tree by enlarging or shrinking the neighbourhood of the co-expressed probe sets. A java application that is able to parse the Newick format produced by the NJ algorithm and export a tree in HTML format was implemented. *r-*value ranked lists of the most closely correlated probe sets, similarly to the first case, are also produced according to the tree hierarchy.

### Over-representation analysis

After a probe set list of the mostly correlated genes to a driver gene is produced by either method, users can view the annotations regarding Gene Names, Gene Descriptions, Biological Processes, Cellular Components, Molecular Functions, EC Numbers, OMIM entries, Pathways, InterPro, or TransFac data. To highlight the overrepresented annotation terms, users can also perform a text-based analysis. HGCA produces summary tables showing the overrepresented terms outlining the most prominent terms of the list, which are trimmed by applying a *p-*value cut-off of 0.05, where the statistical significance of term over-representation is a Benjamini-Hochberg [56] corrected *p*-value which is based on Hypergeometric Distribution [57]:

(6) $$p=1-\sum_{i=k}^{c}\frac{\left(\begin{array}{c} m\\ i \end{array}\right)\left(\begin{array}{c} n-m\\ c-i \end{array}\right)}{\left(\begin{array}{c} n\\ c \end{array}\right)}$$

where *n* is the total number of probe sets, *m* the total number of probe sets that contain the term, *c* is the number of the probe sets of the list and *k* the probe sets of the list that contain the term.

## Results

### Microarray data collection

**Figure 1:**
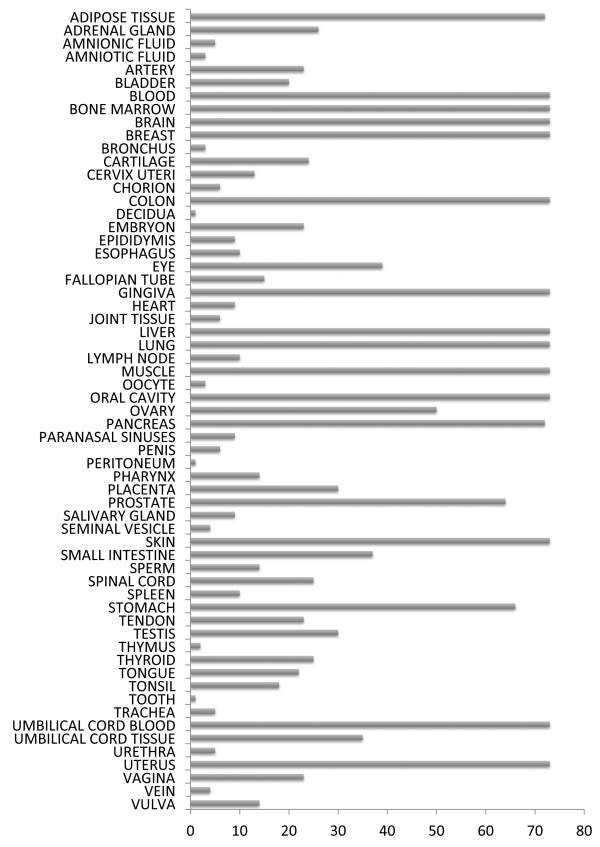
**Tissue/organ GSM distribution.** 1959 microarray samples of normal human tissues were selected from over 62000 samples found in GEO, to maximise the tissue sampling coverage.

62024 GSM SOFT files were parsed, of which only 11599 GSMs met the keyword search criteria. Another 503 GSMs from M^2^DB were also included. After discarding culture cell lines, pathological tissues and false positive control samples 4732 GSMs remained. Tissue/Organ names were manually assigned to those 4732 GSMs of which all probe intensity values were inside acceptable value range, while 280 samples were found by hash algorithms to be duplicates, concluding in a total of 4452 samples divided in 62 different tissues/organs. In order to maximise tissue and GSE sampling coverage, 1959 samples were automatically selected (Figure 1, Additional file 1: Table S1). HGCA coexpression analysis was based on the normalised expression values of those samples.

### Ribosomal proteins

**Figure 2:**
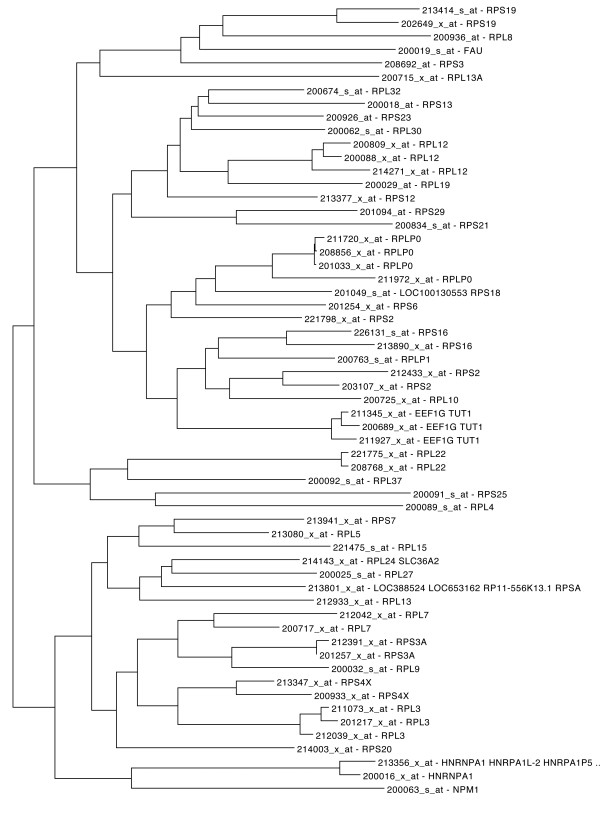
**Tree representation of RPL10 correlated genes.** Tree representation of top ranked correlated genes to RPL10 (ribosomal protein L10) gene, after Neighbour Joining clustering.

Ribosomal proteins are known to be tightly coexpressed in organisms from bacteria to humans [58,59]. We used 200725_x_at probe set which corresponds to the RPL10 (ribosomal protein L10) gene, as HGCA input to the tree tool. A tree of the most closely clustered probe sets to the probe set of interest was constructed (Figure 2). The corresponding genes of the correlated probe sets were mostly annotated as ribosomal proteins, as well. The text analysis of the gene descriptions showed that the term “ribosomal” was found to be over-represented (*p*-value: 1.7e–69). A similar analysis for all aspects of Gene Ontology (Biological Process, Cellular Component and Molecular Function) showed the over-representation of the GO terms GO:0006412 (“translation”) (*p*-value: 7.6e–54), GO:0005840 (“ribosome”) (*p*-value: 2.6e–64) and GO:0003735 (“structural constituent of ribosome”) (*p*-value: 1.7e–67), respectively. Finally, the “Ribosomal_Proteins” Pathway, was also over-represented (*p*-value: 7.5e–49).

An *r-*value-ranked list of the 50 most correlated probe sets to the probe set of interest was produced. The results were comparable with those of the tree-based analysis: 47 of the corresponding genes of the correlated probe sets were annotated as ribosomal proteins, while the remaining two ones were characterised as eukaryotic translation elongation factors. The term “ribosomal” was found to be over-represented (*p*-value: 5.0e–84). Similarely, an over-representation of GO terms was also found: GO:0006412 (“translation”) (*p*-value: 1.4e–70), GO:0005840 (“ribosome”) (*p*-value: 1.5e–77) and GO:0003735 (“structural constituent of ribosome”) (*p*-value: 4.4e–80). Finally, the “Ribosomal_Proteins” Pathway, was also over-represented (*p*-value: 1.5e–56).

### HLA proteins

**Figure 3:**
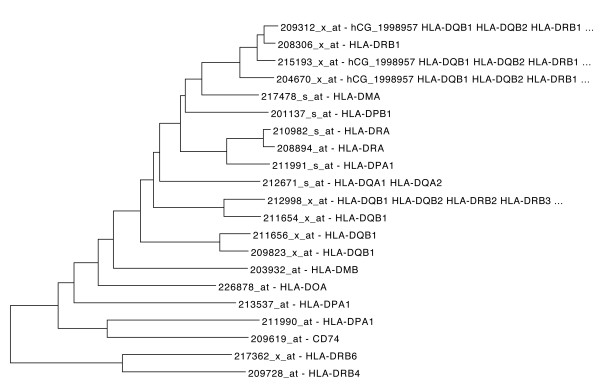
**Tree representation of HLA correlated genes.** Tree representation of top ranked correlated genes to HLA-DOA (major histocompatibility complex, class II, Doα).

Another tightly regulated set of genes are those of HLA family. 226878_at which corresponds to HLA-DOA (major histocompatibility complex, class II, Doα) was used as a driver probe set. Half of the probe sets which correspond to Major Histocompatibility Complex II, are found to be strongly coexpressed (Figure 3). The overrepresentation of GO terms related to that family of proteins is evident: The *p*-values of GO:0002504 (antigen processing and presentation of peptide or polysaccharide antigen via MHC class II), GO:0042613 (MHC class II protein complex) and GO:0032395 (MHC class II receptor activity) are 6.1e–28, 6.1e–29 and 2.3e–26, respectively.

### Metallothionein proteins

**Figure 4:**
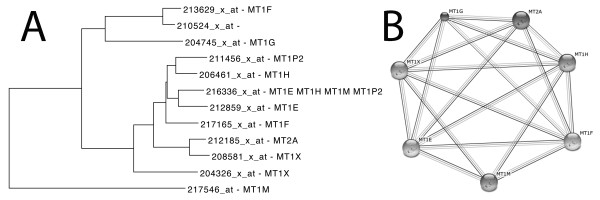
**MT1H coexpressed genes. A**. Tree representation of top ranked correlated genes to MT1H (metallothionein 1 H) gene. **B**. String interactors of MT1H.

Metallothioneins have a high content of cysteine residues that bind various heavy metals. They are transcriptionally regulated by both heavy metals and glucocorticoids [60]. We used the 206461_x_at probe set, which corresponds to the driver gene MT1H (metallothionein 1 H) gene. A tree of the most closely clustered probe sets to it was constructed (Figure 4A). 11 out of 15 probe sets that correspond to metallothionein genes are clustered together. From the remaining 4 probe sets, 2 correspond to pseudogenes. A String [61] analysis, produced similar evidence about the interactors of MT1H (Figure 4B).

### Prostaglandin D2 synthase 21 kDa (brain) coexpressed proteins

**Figure 5:**
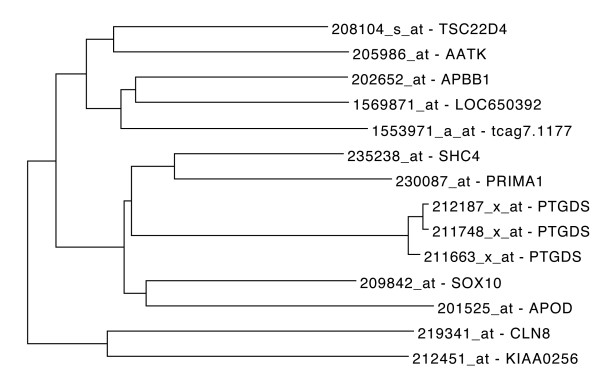
**PTGDS coexpressed genes.** Tree representation of top ranked correlated genes to PTGDS, a gene that is involved in androgenic alopecia. Of particular interest, is SOX10, a gene that is involved in sex development.

Testosterone is necessary for the development of male pattern baldness, known as androgenetic alopecia (AGA) [62]. A very recent study showed that prostaglandin D(2) synthase (PTGDS) is elevated at the mRNA and protein levels in bald scalp compared to haired scalp of men with AGA [63]. 211663_x_at which corresponds to PTGDS was used as driver probe set for an HGCA analysis (Figure 5). Among the coexpressed genes, the transcription factor SOX10 (Sex determining Region Y-box 10) is found. As the role of SOX10 in human 22q-linked disorders of sex development is implied by the fact that transgenic SOX10 expression in gonads of XX mice resulted in development of testes and male physiology [64], the relation of SOX10 to AGA would be worth exploring. Furthermore, another 3 unknown gene loci (LOC650392, tcag7.1177 and KIAA0256) which are coexpressed with PTGDS could be of interest.

### DDX4 coexpressed proteins

**Figure 6:**
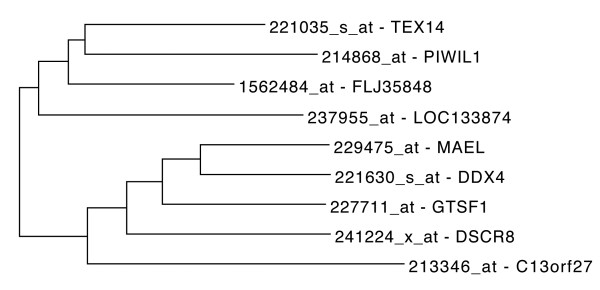
**Tree representation of DDX4 correlated genes.** Tree representation of top ranked correlated genes to DDX4 gene. Coexpressed DDX4, MAEL and PIWIL1 proteins are known to interact with each other.

DEAD box polypeptide 4 (DDX4) belongs to the DEAD box family of putative RNA helicases which are characterised by the conserved DEAD (Asp-Glu-Ala-Asp) motif. DDX4 encodes a protein, which is a homolog of VASA proteins in *Drosophila* and several other species. It is specifically expressed in the germ cell lineage in both sexes and functions in germ cell development. HGCA analysis using 221630_s_at probe set (Figure 6), revealed that DDX4 is coexpressed with MAEL and PIWIL1 genes. DDX4 protein interacts with PIWIL1 protein in mouse testis and 293T cells [65]. DDX4 protein interacts with MAEL which also interacts with PIWIL1 [66] in mice. Another coexpressed gene, is TEX14 (testis expressed 14) which is required in spermatogenesis and male fertility. In HGCA analysis, the term “GAMETOCYTE” was over-represented (*p*-value 4.6e–3).

## Discussion

The probe set lists produced by ranking or clustering can be used to explore genes that may potentially participate in similar biological processes other than the known genes. Alternatively, the annotation over-representations can be used to predict potential biological functions of genes. Our term-counting tool provides a statistical basis for interpreting such themes. Similarly, analysis of the promoters of a set of coexpressed genes for over-represented motifs may give confidence in transcription factor-binding site prediction which would not be possible by comparison of a single promoter against a database of known motifs.

As opposed to previous attempts [12,13,15], our tool, Human Gene Correlation Analysis (HGCA), performs a full scale clustering analysis of a large human microarray dataset. Human Gene Coexpression Landscape (HGCL) [12] uses duplicates of 24 human tissue samples. Although Coexpression Analysis of Human Genes (CAHG) [13] uses ~4000 microarrays, they belong to 60 out-dated datasets from various platforms. The number of microarrays of each dataset ranges from 10 to 255 where only 4 of them exceed 130 samples. The current study uses a single dataset of ~2000 microarrays of a single platform and the same normalisation procedure. Data uniformity and large dataset size increase the statistical significance of our results. Although all three studies start with an all-against-all weighted graph where each vertex represents a probe and the weight of each edge the coexpression level between a pair of vertices, the other two studies eliminate the majority of edges by applying a threshold to produce unweighted graphs, resulting in information content loss. To compensate, they reintroduce weights on the remaining edges based either on their observation frequency (CAHG) or their intersection of the results of two algorithmic approaches (HGCL). On the contrary, to produce a hierarchical network representation, our approach leaves the weighted graph intact. By doing so, the ability to find anti-correlated genes in the ranked list approach is also preserved. Another source of information loss is the exclusion of cross-hybridizing probe sets (probe sets that recognise more than one gene). Although this approach increases specificity, it reduces coverage and thus, potential hypotheses cannot be explored. In the case of HCGL excluding the two-element clusters produces further coverage reduction. For those reasons, although the results of HGCL and ours are generally comparable, our tool performs better in the detection of co-clustered genes. For example in the metal-ion homeostasis cluster HGCL failed to detect MT1P2, in histocompatibility complex it failed to detect HLA-DOA, HLA-DQB1, HLA-DQB2, HLA-DRB2, HLA-DRB3, HLA-DQA2, etc. In our analysis, all term overrepresentations are supported by an indication of statistical significance. In HGCL, external algorithms offer such analysis, a missing option in CAHG. GEO datasets are analysed using combinations of three different distances (Uncentered Correlation, Pearson Correlation, Euclidean) and three clustering algorithms (UPGMA, Single Linkage, Complete Linkage). Nevertheless, the use of Euclidean distances or the Single Linkage hierarchical approach, fails to cluster genes meaningfully due to the production of over-heighted trees. The best combination is the use of Pearson Correlation and UPGMA hierarchical clustering. On the contrary HGCA uses NJ, an algorithm that attempts to correct the UPGMA method for its assumption that the same distance rate applies to each probe set. NJ outperforms all other distance-based clustering algorithms in simulation studies [67] and it is the most computationally efficient. While GEO’s visualisation tool provides intuitive snapshots, it is not highly interactive and user friendly and therefore one is difficult to detect a gene of interest. Although HGCA comes with an integrated tree viewer, it is currently able to export the clustering hierarchy in Newick format to be browsed by higher quality external tree viewers [55]. In addition, both GEO and Gemma [14] tools lack term overrepresentation analysis. Finally, while our tool is based on MAS5.0-Pearson combination, an approach which was also employed by successful tools such as ACT or Expression Angler, the use of MAS5.0-Spearman or RMA-Pearson combinations proposed by HGCL is debatable [8].

BioGPS [23] is a data integration platform yet a powerful a tool to explore neighbour genes based on their expression profiles. It has significant differences compared to HGCA which make the two applications complementary to each other. While BioGPS is based on duplicates of 88 human tissue samples, HGCA is based on almost 2000 samples from many tissues. Furthermore, BioGPS uses HG-U133A chip while HGCA uses HG-U133 Plus 2 which is comprised of more than double probe sets (~22000 vs ~55000). Finally, BioGPS lacks clustering and over-representation analysis.

WGCNA [16] is a collection of R functions for network analysis. In terms of data visualisation and interactivity, WGCNA comes with static images to present data clustering which can be exported to Cytoscape. As it can only export one session per time, this is tedious and time consuming. On the other hand, HGCA does not handle data at a network level, but at a tree hierarchy and it comes with its own embedded interactive tree viewer. To gain in functionality, HGCA also exports clusters in Newick tree file format to present data using external viewers such as Dendroscope [68] or iTOL [69]. In addition, while WGCNA is restricted to R experts, HGCA comes with a web interface, that experimentalists are familiar with. Finally, WGCNA does not provide any over-representation analysis which is the scope of HGCA.

BioLayout Express^3D^[24] is an advanced visualisation standalone application able to analyse biological networks in 3D. Related studies, such as [70] take advantage of its rich functionality to construct, visualise, and cluster transcription networks from microarray expression data. While HGCA analyses data using a hierarchical approach and not at a network level, BioLayout Express^3D^ is memory inefficient and computationally expensive to cope with HGCA’s data complexity. In order to address the issue of data scaling, an advance graphics card is necessary. Finally while MCL clustering [71] is provided within BioLayout Express^3D^ application, HGCA uses the NJ algorithm to cluster data in a hierarchy.

## Conclusions

HGCA is an attractive, easy-to-use and powerful tool for the discovery of genes that are associated in related functions based on their coexpression patterns and thus it is a competitive and yet useful knowledge discovery web based tool for experimentalists.

## Availability and requirements

**Projects Home Page**: http://biobank-informatics.bioacademy.gr/coexpression

**Programming Language**: Written in C, PHP, Java and MySQL

**Requirements**: Any web browser. HTML source code is validated by W3 validator according to HTML 4.01 Transitional DTD.

## Competing interests

The authors declare that they have no competing interests.

## Authors’ contributions

Wrote the paper: IM and GAP. Designed and implemented the web site and database: IM. Contributed analysis tools: GAP, AM, MAK and AK. Clustering analysis and tree visualisation: GAP. Provided computational support and contributed to the critical revision of the manuscript: RS. Provided guidance: SK. All of the authors have read and approved the manuscript.

## Supplementary Material

Additional file 1 **List of the GSM samples which were used in the analysis.** The titles of the GSM samples and their corresponding GSE series, as well as their tissue/organ names are shown. Click here for file
